# Molecular Network *Polyamorphism* in Mechanically Activated Arsenic Selenides Under Deviation from As_2_Se_3_ Stoichiometry

**DOI:** 10.3390/molecules30030642

**Published:** 2025-01-31

**Authors:** Oleh Shpotyuk, Zdenka Lukáčová Bujňáková, Peter Baláž, Yaroslav Shpotyuk, Malgorzata Hyla, Andrzej Kozdras, Adam Ingram, Vitaliy Boyko, Pavlo Demchenko, Andriy Kovalskiy

**Affiliations:** 1 Institute of Physics, Jan Dlugosz University in Częstochowa, 13/15, al. Armii Krajowej, 42-200 Częstochowa, Poland; m.hyla@ujd.edu.pl; 2 O.G. Vlokh Institute of Physical Optics, Ivan Franko National University of Lviv, 23, Dragomanov Str., 79005 Lviv, Ukraine; boykovit@gmail.com; 3 Institute of Geotechnics of Slovak Academy of Sciences, 45, Watsonova Str., 04001 Košice, Slovakia; bujnakova@saske.sk (Z.L.B.); balaz@saske.sk (P.B.); 4 Department of Sensor and Semiconductor Electronics, Ivan Franko National University of Lviv, 107, Tarnavskoho Str., 79017 Lviv, Ukraine; yashpotyuk@gmail.com; 5 Institute of Physics, University of Rzeszow, 1, Pigonia Str., 35-959 Rzeszow, Poland; 6 Faculty of Physics, Opole University of Technology, 75, Ozimska Str., 45-370 Opole, Poland; a.kozdras@po.edu.pl (A.K.); a.ingram@po.edu.pl (A.I.); 7 Department of Inorganic Chemistry, Ivan Franko National University of Lviv, 6, Kyryla i Mefodiya Str., 79000 Lviv, Ukraine; pavlo.demchenko@lnu.edu.ua; 8 Department of Physics, Engineering and Astronomy, Austin Peay State University, Clarksville, TN 37044, USA; kovalskiya@apsu.edu

**Keywords:** thioarsenide molecules, polyamorphism, polyamorphic transition, arsenoselenides, x-ray diffraction, Raman microspectroscopy, positron annihilation lifetime, thermal analysis, cluster modelling, reamorphization

## Abstract

Polyamorphic transitions driven by high-energy mechanical milling (nanomilling) are studied in thioarsenide As_4_Se_n_-type glassy alloys obtained by melt quenching deviated from arsenic triselenide As_2_Se_3_ stoichiometry towards tetraarsenic pentaselenide (g-As_4_Se_5_) and tetraarsenic tetraselenide (g-As_4_Se_4_). This employs a multiexperimental approach based on powder X-ray diffraction (XRD) analysis complemented by thermophysical heat transfer, micro-Raman scattering (micro-RS) spectroscopy, and revised positron annihilation lifetime (PAL) analysis. Microstructure scenarios of these nanomilling-driven transformations in arsenoselenides are identified by quantum-chemical modeling using the authorized modeling code CINCA (the Cation Interlinked Network Cluster Approach). A straightforward interpretation of a medium-range structure response of a nanomilling-driven polyamorphism in the arsenoselenides is developed within the modified microcrystalline model. Within this model, the diffuse peak-halos arrangement in the XRD patterning is treated as a superposition of the Bragg-diffraction contribution from inter-planar correlations supplemented by the Ehrenfest-diffraction contribution from inter-atomic (inter-molecular) correlations related to derivatives of network As_2_Se_3_-type and molecular As_4_Se_4_-type conformations. Changes in the medium-range structure of examined glassy arsenoselenides subjected to nanomilling occur as an interplay between disrupted intermediate-range ordering and enhanced extended-range ordering. The domination of network-forming conformations in arsenoselenides deviated from As_2_Se_3_ stoichiometry (such as g-As_4_Se_5_) results in rather slight changes in their calorimetric heat-transfer and micro-RS responses. At the atomic-deficient level probed by PAL spectroscopy, these changes are accompanied by reduced positron trapping rate of agglomerated multiatomic vacancies and vacancy-type clusters in an amorphous As-Se network. Under an increase in As content beyond the g-As_4_Se_5_ composition approaching g-As_4_Se_4_, nanomilling-driven polyamorphic transitions, which can be classified as reamorphization (amorphous I-to-amorphous II) phase transitions, are essentially enhanced due to the higher molecularity of these glassy alloys enriched in thioarsenide-type As_4_Se_4_ cage-like molecular entities and their low-order network-forming derivatives.

## 1. Introduction

Arsenic selenides As_x_Se_100-x_ (*arsenoselenides*) compose an important class of substances with saturated covalent bonding, which could be stabilized in a glassy (g) state within the compositional domain from ‘pure’ g-Se (*x* = 0) to As-rich (~70–75 at. % As) species [1,2,3,4]. The stoichiometric arsenic triselenide g-As_2_Se_3_ (*x* = 40) is known to be a principal network glass-former in this canonical glassy system [2]. Under minor deviation from As_2_Se_3_ stoichiometry in the Se-rich side (x ≤ 40), these glasses exhibit a layered- or chain-type network arrangement [2,3], which is practically insensitive to post-technological modification [5]. That is why on the path tailoring special vitreous arsenoselenides, the most attractive seems to be the latter domain comprising the As-bearing alloys beyond As_2_Se_3_ stoichiometry (40 < *x* < ~75), which are enriched in so-called thioarsenide As_4_Se_n_-type covalently bonded cage molecular entities (where *n* is an integer less than 6, accepting a whole family of *arsenoselenides*, the values are 4, 3, and 0 [2,3,6,7,8]). Herein, the *thioarsenide* nomenclature As_4_X_n_ introduced initially for arsenic sulfides under the analysis of molecular packing conformations by Bonazzi and Bindi [9] and electron density distributions by Gibbs et al. [10] is used by analogy for *arsenoselenides*.

Under the deviation from As_2_Se_3_ stoichiometry in the over-stoichiometric As-rich side, the phase diagram of the As-Se system [11] contains three stable compounds. These are stoichiometric arsenic triselenide As_2_Se_3_ (compositionally equivalent in *thioarsenide* nomenclature to tetraarsenic hexaselenide As_4_Se_6_, n = 6) showing not a molecular but a layered network-crystalline arrangement [2,3,4]), and two molecular crystalline compounds such as tetraarsenic tetraselenide As_4_Se_4_ (equivalent to arsenic monoselenide AsSe [7]) and tetraarsenic triselenide As_4_Se_3_ [8]. Thus, in accordance with [3,7,8], a full diversity of thioarsenide As_4_Se_n_-type molecular crystalline structures in the As-Se system is restricted by monoclinic As_4_Se_4_ (n = 4), orthorhombic As_4_Se_3_ (n = 3), and rhombohedral As_4_ (n = 0). Metastable alternatives are only possible among As_4_Se_3_ polymorphs due to phase equilibria in the vicinity of this composition [8]. Indeed, as was reported by Blachnik and Wickel [12], under heating above 412 K, the ambient-temperature monoclinic α-As_4_Se_3_ transforms in high-temperature orthorhombic α’-As_4_Se_3_, and under heating above 447K, the latter transforms into plastically-crystalline β-As_4_Se_3_ phase and an unidentified amorphous substance; only a normally crystalline orthorhombic α’-As_4_Se_3_ phase could be stabilized in a metastable form by quenching. Therefore, in the case of a molecular crystalline *As_4_Se_3_ thioarsenide* composition, we expect *polymorphism* due to inter-crystalline (crystalline I–to–crystalline II) transitions. In contrast, a *polyamorphism* (in terms of pressure-induced molecular–to–network transition in chalcogenide glasses [13,14]) is a character for As-rich *arsenoselenides* As_x_Se_100-x_ (x > 40) composed of network derivatives from As_4_Se_n_ molecules whenever their composition is defined by *n* parameter. In reality, *polyamorphic* transformations occur due to a variety of amorphous conformations that emerged through amorphization (crystalline–to–amorphous) or reamorphization (amorphous I–to–amorphous II) phase transitions, which could be activated, in part, by high-energy mechanical milling also termed as nanomilling [7,8].

The scope of this research is to justify the most plausible microstructural scenarios of nanomilling-driven polyamorphic transitions in glassy arsenoselenides g-As_x_Se_100-x_ (*x* > 40) under small deviations from network-structured stoichiometric g-As_2_Se_3_ towards over-stoichiometric molecular network compounds approaching thioarsenide As_4_Se_n_-type ones like tetraarsenic pentaselenide As_4_Se_5_ (n = 5) and tetraarsenic tetraselenide As_4_Se_4_ (n = 4). With this aim, the multi-experimental characterization probes including powder X-ray diffraction (XRD) in terms of modified microcrystalline model [5,6,7,8], micro-Raman scattering (micro-RS) spectroscopy [7,8], calorimetric heat-transfer studies employing temperature modulated DSC-TOPEM method [8], and revised positron annihilation lifetime (PAL) analysis [15], complemented with ab initio quantum-chemical modeling using the authorized atomic cluster-simulation code CINCA (the Cation-Interlinked Network Cluster Approach) [16] have been employed.

## 2. Results and Discussion

### 2.1. Nanostructurization-Driven Medium-Range Structural Changes in Glassy Arsenoselenides Under Minor Deviation from As_2_Se_3_ Stoichiometry

**Figure 1:**
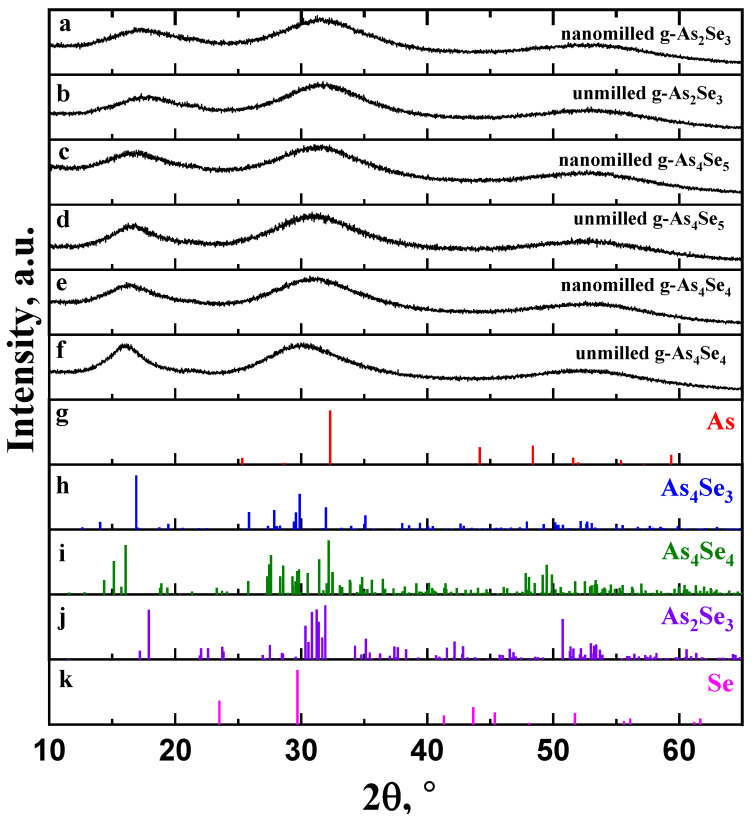
The XRD patterning in unmilled and nanomilled g-As_2_Se_3_ (**a**,**b**), g-As_4_Se_5_ (**c**,**d**), and g-As_4_Se_4_ (**e**,**f**) showing diffuse peak-halos corresponding to the FSDP (~15–25°2*θ*), SSDP (~28–33°2*θ*) and TDP (~50–60°2*θ*) in comparison with the Bragg-diffraction reflexes from crystalline phases: (**g**) trigonal Se (JCPDS No. 73-0465), (**h**) monoclinic As_2_Se_3_ (JCPDS No. 65-2365), (**i**) monoclinic As_4_Se_4_ (JCPDS No. 71-0388), (**j**) orthorhombic As_4_Se_3_ (JCPDS No. 04-4979), and (**k**) rhombohedral As (JCPDS No. 72-1048).

The XRD patterns collected for glassy arsenoselenides of stoichiometric g-As_2_Se_3_ and over-stoichiometric thioarsenide-type g-As_4_Se_5_ and g-As_4_Se_4_ samples before and after nanomilling superimposed with the Bragg-diffraction reflexes from prominent crystalline phases in binary As-Se system, such as trigonal Se (JCPDS No. 73-0465) [17], monoclinic As_2_Se_3_ (JCPDS No. 65-2365) [18,19], monoclinic As_4_Se_4_ (JCPDS No. 71-0388) [19,20,21], orthorhombic As_4_Se_3_ (JCPDS No. 04-4979) [22] and rhombohedral As (JCPDS No. 72-1048) [23,24], are reproduced in Figure 1.

The XRD patterns reproduced in Figure 1 clearly demonstrate a so-called three-peak structure of glassy arsenoselenides [5,6,7,8,25,26] composed of separated peak-halos reproduced in the structure factor of these glasses as the first sharp diffraction peak (FSDP), located at diffraction angles of ~15–25°2*θ* corresponding to scattering vectors in a reciprocal space *Q_1_* ≅ ~1.0–1.5 Å^−1^), the second sharp diffraction peak (SSDP) at ~28–33°2*θ* and *Q_2_*~1.8–2.2 Å^−1^, and the third diffraction peak (TDP) at ~50–60°2*θ* and *Q_3_*~3.3–4.0 Å^−1^. For glassy networks composed of directional units, the FSDP and SSDP are narrowed in a width of *ΔQ*_1,2_ < ~0.3–0.4 Å^−1^. The FSDP is commensurable with intermediate-range ordering (IRO) of some structural entities at the lengths approaching tens Å, and SSDP is associated with extended-range ordering (ERO) related to the sizes of these entities [25,26,27,28]. According to the Fourier-transform analysis [26], each of these peak-halos interpreted through the Bragg diffraction formalism corresponds to real-space ordering with effective periodicity R and correlation length L. In contrast, the TDP is ascribed to the shortest nearest-neighbor separation in a glass insensitive to medium-range structure [25].

The parameters of the FSDP-related peak-halos in unmilled and nanomilled glassy arsenoselenides under deviation from As_2_Se_3_ stoichiometry in the As-rich side are gathered in Table 1. With this trend around As_4_Se_5_ composition, the FSDP becomes sharper owing to strong narrowing in a width Δ*Q_1_* (Figure 1), resulting in prolonged correlation lengths *L*. Simultaneously, the FSDP shifts to lower *Q_1_* values resulting in enlarged distances *R*. Similar features are characteristic for the SSDP (see, [6]), speaking in favor of growing molecularity in As-rich arsenoselenides. Being subjected to nanomilling, these glasses return closer to network-type conformations, becoming more broadened in the FSDP- and SSDP-related peak-halos shifted to higher diffraction angles (see Table 1).

Within the modified microcrystalline model [5,6,7,8], the FSDP in glassy arsenoselenides can be considered as diffuse peak-halos originating from the overlapped inter-planar and inter-molecular (-atomic) correlations ascribed to some remnants of molecular and network crystalline structures. Thus, the FSDP position in g-As_4_Se_5_ (Figure 1a) near *Q_1_*~1.19 Å^−1^ (respectively, corresponding to inter-planar and inter-atomic distances *R*~5.28 Å and *d_s_*~6.50 Å) is found to be in an excellent agreement with the Bragg-diffraction lines arising from the (111) plane (equiv. *R*~5.243 Å) in orthorhombic As_4_Se_3_ [22] and the (120) plane (equiv. *R*~5.512 Å) in monoclinic As_4_Se_4_ [19,20,21]. In contrast, the diffraction line arising from the (020) plane (equiv. *R*~5.512 Å) in layer-type monoclinic As_2_Se_3_ [18,19] is shifted to higher diffraction angles. This observation confirms mixed molecular network structural conformations in glassy arsenoselenides deviated from As_2_Se_3_ stoichiometry.

As can be inferred from Figure 1, nanomilling does not alter diffuse peak-halos in the XRD patterning of g-As_4_Se_5_, testifying in favor of polyamorphism in this glass due to the nanomilling-driven (amorphous I–to–amorphous II) transition known as the reamorphization transition [6,7,8]. In nanomilled samples of g-As_4_Se_5_, the FSDP loses intensity, becomes more weakened, shifts to higher scattering vectors *Q*_1_ (from 1.19 Å^−1^ to ~1.21 Å^−1^), and broadens in width Δ*Q*_1_ (from 0.30 Å^−1^ to ~0.38 Å^−1^). Respectively, the spacing of the FSDP-responsible quasi-periodicity *R* slightly decreases (from 5.28 to ~5.18 Å), while the correlation length *L* (over which this quasi-periodicity is maintained) decreases more gradually (from 21.02 to ~16.33 Å); thus, nanomilling-driven fragmentation of the FSDP-responsible medium-range structural entities results in disrupted IRO. The similar, albeit more reduced, changes occur in the SSDP (see also [5]), signaling that nanomilling-driven fragmentation of structural entities is responsible for this diffuse peak-halo; this effect is accepted (under current normalization procedure in the XRD analysis) as a signature of increased ERO.

Thus, inter-molecular and inter-atomic correlations responsible for IRO are merely destroyed under nanomilling (resulting in disrupted IRO) at the cost of inter-planar correlations (resulting in increased ERO), shifting g-As_4_Se_5_ closer to layer-type network-crystalline stoichiometric g-As_2_Se_3_. Microstructurally, this process is a clear manifestation of the interplay between IRO and ERO in a molecular network arsenoselenide g-As_4_Se_5_ undergoing nanomilling-driven reamorphization towards a more network-structured state. Such changes are better refined in thioarsenide As_4_Se_n_-type arsenoselenides with n = 4 (that is, glassy arsenic monoselenide g-AsSe), evidently dominated in the unmilled state of this glass by molecular-type conformations (see Figure 1e). As a consequence of high molecularity, the nanomilling-driven molecular-to-network reamorphization transition in g-As_4_Se_4_ is accompanied by a stronger response in medium-range structures (see Table 1), also revealed as an interplay between IRO and ERO [7].

### 2.2. Thermophysical Heat Transfer and Micro-RS Response in Glassy Arsenoselenides Under Minor Deviation from As_2_Se_3_ Stoichiometry

**Figure 2:**
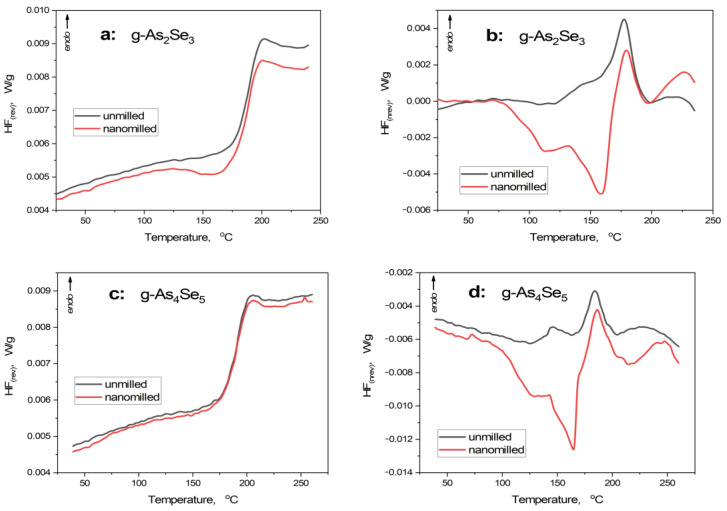
Modulated DSC-TOPEM profiles showing temperature variation of reversing *HF*_rev_ (**a**,**c**) and non-reversing *HF*_nrev_ (**b**,**d**) heat flow in unmilled (black curve) and nanomilled (red curve) stoichiometric g-As_2_Se_3_ (**a**,**b**) and over-stoichiometric As-rich glass approaching g-As_4_Se_5_ (**c**,**d**).

Thermoanalytical heat transfer responses in nanomilling-driven transformations in arsenoselenides are summarized by the DSC-TOPEM profiles reproduced in Figure 2. The calorimetric parameters specifying the behavior of reversing (*HF*_rev_) and non-reversing (*HF*_nrev_) heat flow curves derived from these profiles are presented in Table 2.

Under the first heating run, the principal endothermic thermal-alteration event represents glass transition [29,30,31]. This stepwise calorimetric heat-transfer phenomenon is compositionally dependent on glassy chalcogenides possessing mixed molecular network conformations (see [8,32]).

In g-As_2_Se_3_, the sharp step-like jump is revealed in the temperature dependence of *HF*_rev_, creating heat capacity variation Δ*C*_p_ of 0.20 J·g^−1^·K^−1^ and onset glass transition temperature *T*_g_^onset^ of 179.0 °C (see Figure 2a, Table 2), in good respect to [33,34]. In *HF*_nrev_ dependence (Figure 2b), this *endothermic* event is revealed as a distinct peak close to *T*_g_, creating specific enthalpy differences Δ*H* of 6.7 J·g^−1^ (slight asymmetry of this peak is caused by the coarse-grained microstructure of this glass). Nanomilling does not change the principal heat transfer phenomena in g-As_2_Se_3_ as it follows from the step-like jump in *HF*_rev_, causing it to be nearly the same as other parameters (such as Δ*C*_p_ ~ 0.20 J·g^−1^·K^−1^ and *T*_g_^onset^ ~ 178.3 °C), although with a distinct pre-*T*_g_ exotherm due to relaxation of inner stress under nanomilling. In *HF*_nrev_ determination (see Figure 2b), the changes are more pronounced, being revealed as a broad double-well exotherm due to relaxation of strong inner stress (before nearly the same endothermic peak), resulting in negative Δ*H* approaching -10.9 J·g^−1^ (see Table 2).

Such temperature behavior characteristics for layered network glasses like g-As_2_Se_3_ are neatly invariant under deviation from chemical stoichiometry towards g-As_4_Se_5_ (see Figure 2c,d and Table 2). The only difference concerns the absence of a pre-*T*_g_ exotherm in the *HF*_rev_ curve (Figure 2c), testifying to a more homogeneous network structure of this glass stabilized under nanomilling as compared with g-As_2_Se_3_. Exploring the concept of mixed molecular network structures of over-stoichiometric As-rich arsenoselenides [5,6,7,8], this specificity could be ascribed to the nanomilling-driven improvement of a layer-type network structure of this glass rather than destroyed thioarsenide-type molecules.

**Figure 3:**
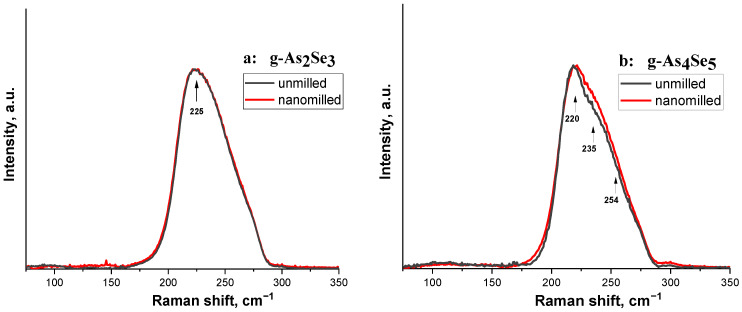
The normalized micro-RS spectra collected from nanomilled (red curve) and unmilled (black curve) samples of g-As_2_Se_3_ (**a**) and As-rich samples compositionally approaching g-As_4_Se_5_ (**b**). The character features in micro-RS spectra of the examined samples are distinguished by arrows.

The preference for network-type conformations in the structure of arsenoselenide glasses is also confirmed by micro-RS responses compared to unmilled and nanomilled samples of stoichiometric g-As_2_Se_3_ and over-stoichiometric g-As_4_Se_5_ (Figure 3a,b).

The micro-RS spectra of unmilled stoichiometric g-As_2_Se_3_ (see Figure 3a, black curves) show a very strong and broad band near ~220–230 cm^−1^ signalizing on the structural network of this glass built of corner-sharing AsSe_3/2_ pyramids [35,36]. Under minor deviation from As_2_Se_3_ stoichiometry towards g-As_4_Se_5_, only a few slight features are revealed at the background of this strong vibrational band (see Figure 3b, black curve).

After nanomilling, the micro-RS response in both samples (g-As_2_Se_3_ and g-As_4_Se_5_) becomes smoother (see Figure 3a,b, red curves), presumably because of the transition to a more perfect network structure without a notable amount of thioarsenide molecules and molecular entities as was in more As-enriched arsenoselenides [7,8].

Thus, subjected to nanomilling, the g-As_4_Se_5_ is transferred towards network conformations with a negligible or very small content of thioarsenide-type molecules.

### 2.3. Atomic-Deficient Microstructure of the Examined Glassy Arsenoselenides

**Figure 4:**
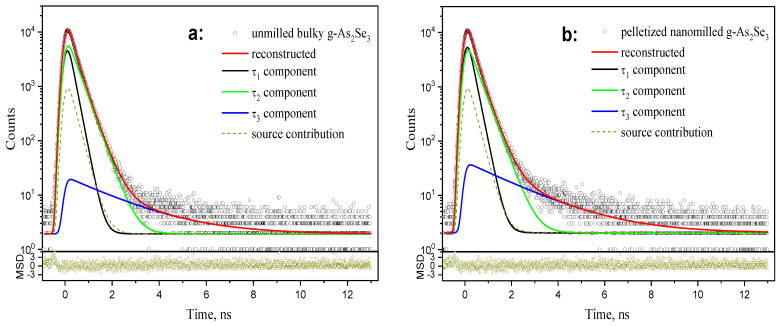
The raw PAL spectra of bulky-unmilled (**a**) and pelletized-nanomilled (**b**) glass samples of stoichiometric g-As_2_Se_3_ reconstructed from unconstrained three-term fitting (depicted at the background of source contribution and bottom insets showing statistical scatter of variance).

The PAL spectra reconstructed from unconstrained three-term fitting for bulk discs of melt-quenching derived stoichiometric g-As_2_Se_3_ and disc-shaped pellets prepared from this glass subjected to nanomilling are reproduced in Figure 4a,b.

Similar PAL spectra were also collected for other arsenoselenide samples that deviated from As_2_Se_3_ (compositionally approaching g-As_4_Se_5_ and g-As_4_Se_4_). The best-fit parameters (lifetimes *τ_i_* and intensities *I_i_*, *i* = 1,2,3) are gathered in Table 3, and the positron trapping modes derived from these PAL spectra within canonical two-state simple trapping model [15], ignoring the contribution of decaying bound positron-electron (positronium, Ps) states, are presented in Table 4.

The PAL spectra of examined arsenoselenides can be decomposed on three free components with the most decisive input from the positron-trapping channel because of negligible Ps-decaying contribution (indeed, the third component intensity *I*_3_ does not exceed 1.5% in any of the samples in Table 3). It means that positron annihilation is preferred by trapping from free-volume defect states, and, therefore, this process can be adequately parametrized employing a two-state simple trapping model, ignoring Ps-decaying from holes with a radius of *R_3_*~0.3 nm and a fractional free volume *f_v_* < ~0.3 (see Table 4) [15].

Compositional trends in average positron lifetimes (*τ_av_*) and molar volume (derived from atomic densities) of glassy arsenoselenides around As_2_Se_3_ stoichiometry do not coincide. This can be explained in terms of the decisive role of positron traps identified as free-volume voids built of intrinsic bond-free solid angles around constituent atoms with an effective negative electrical charge at the constituent chalcogen atoms [15,37]. As a result, the PAL spectra of these glasses show a broad maximum in *τ_av_* near stoichiometric g-As_2_Se_3_ supplemented by slight steps and some irregular jumps in defect-specific lifetimes *τ_2_* in over-stoichiometric As-rich glasses. These irregularities are suggested to be related to some deviations in molecular network conformations of these over-stoichiometric glasses, approaching thioarsenide As_4_Se_n_-type compositions [7,8].

As follows from Table 3, the PAL spectra of unmilled g-As_2_Se_3_ and g-As_4_Se_5_ samples reveal nearly the same average and defect-specific positron lifetimes (*τ_av_*~ 0.310 ns and *τ_2_*~ 0.358 ns), notably changed only in g-As_4_Se_4_ (increased to *τ_av_*~ 0.322 ns and *τ_2_*~ 0.370 ns). This means that the molecularity of the As_4_Se_5_-bearing thioarsenide is hidden by preferential network-forming tendencies in these glasses while being better revealed in g-As_4_Se_4_. Specifically to arsenoselenide glass chemistry, these defect lifetimes of *τ_2_*~ 0.36–0.38 ns, along with defect-free bulk positron lifetimes of *τ_b_*~ 0.27 ns, resulting in a *τ_2_/τ*_b_ ratio approaching as high as ~1.3–1.4 (see Table 4), are characteristic for multiatomic (bi, tri, or even quadruple) vacancies in an amorphous As-Se matrix [15,37].

Under nanomilling, such relatively large positron-trapping voids become agglomerated, becoming a more favorable environment for volumetric growing (leading to increased *τ_2_* lifetimes) by their merging (accompanied by decreased *I*_2_ intensities), thereby resulting in a reduced positron trapping rate in defects *κ*_d_ and the fraction of trapped positrons *η*. This trapping modification process, referred to as void agglomeration II [15], prevails in arsenoselenides of preferential network-type conformations compositionally close to arsenic triselenide (such as g-As_4_Se_5_) while being depressed in arsenoselenides of preferential molecular-type conformations (such as g-As_4_Se_4_), possessing highly agglomerated intrinsic free-volume voids acting as efficient positron-trapping sites (see Table 4).

Thus, under small deviation from As_2_Se_3_ stoichiometry in the As-rich side towards g-As_4_Se_5_, the atomic deficient structure of the examined glassy arsenoselenides merely keeps some features of preferential network-forming structures. The thioarsenide As_4_Se_n_-type molecular species prevail in over-stoichiometric glassy arsenoselenides beyond the As_4_Se_5_ composition under further movement in the As-rich side towards g-As_4_Se_4_.

### 2.4. Network- vs. Molecular-Forming Clustering in Nanostructured Glassy Arsenoselenides Under Small Deviation from As_2_Se_3_ Stoichiometry

As a reference in CINCA modeling of near-stoichiometric clusters in arsenoselenides, we used an optimized configuration of a single AsSe_3/2_ pyramid sharing three Se atoms with neighbors. The calculated cluster-forming energy of this unit (−72.309 kcal/mol [16]) is used to normalize the forming energies (*E_f_*) of other atomic clusters.

**Figure 5:**
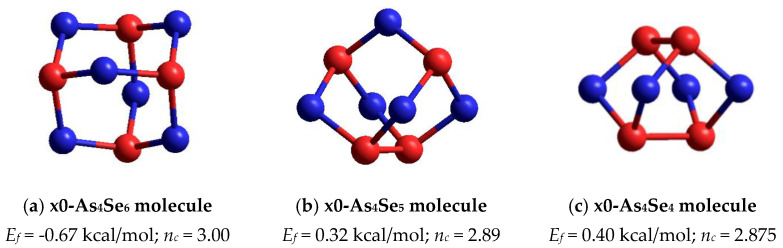
The ball-and-stick presentation of thioarsenide-type cage molecules in vicinity of As_2_Se_3_ stoichiometry: (**a**) tetraarsenic hexaselenide (x0-As_4_Se_6_), (**b**) tetraarsenic pentaselenide (x0-As_4_Se_5_) and (**c**) tetraarsenic tetraselenide (x0-As_4_Se_4_). The Se and As atoms are, respectively, blue- and red-colored, and bonds between atoms are denoted by colored sticks, respectively.

In the point of arsenic triselenide chemical stoichiometry (As_2_Se_3_) corresponding to mean coordination number CN = 2.40 (under the condition of full saturation of covalent bonding appropriate for chalcogenide glasses [1,2,3,4]), we deal with a layer-type network-forming cluster which can be considered as two corner-sharing AsSe_3/2_ pyramids [16]. This cluster possessing forming energy *E_f_* = 0.31 kcal/mol (with respect to AsSe_3/2_ unit) is energetically the most favorable among all layer-type clusters in a binary As-Se system [16]. The calculated structural parameters of this cluster (directly bonded distances and bond angles) are in good agreement with those characteristics for monoclinic As_2_Se_3_ [18,19]. Topologically, this network-forming cluster is optimally constrained, having the number of mechanical constraints per atom of *n_c_* = 3.00 (corresponding to space dimensionality, 3D). Notably, other network clusters reconstructed from neighboring AsSe_3/2_ pyramids (edge- and face-sharing) are impossible in view of very unfavorable *E_f_* energies [16]. The same concerns the thioarsenide As_4_Se_n_-type molecular prototype of this cluster corresponding to n = 6 (that is, a tetraarsenic hexaselenide As_4_Se_6_ molecule composed of four corner-sharing AsSe_3/2_ units in optimally-constrained topology, *n_c_* = 3.00), possessing very unfavorable *E_f_* energy approaching only −0.67 kcal/mol (see Figure 5a).

Under deviation from As_2_Se_3_ stoichiometry on the As-rich side, the first thioarsenide As_4_Se_n_-type molecule is a tetraarsenic pentaselenide As_4_Se_5_ one (corresponding to n = 5). In a family of arsenic sulfides, such a molecule of C_2v_ symmetry is a basic “building” unit of orthorhombic As_4_S_5_ having a natural counterpart known as uzonite [9]. However, there are no molecular crystalline species of this composition (As_4_Se_5_) in the As-Se system.

**Figure 6:**
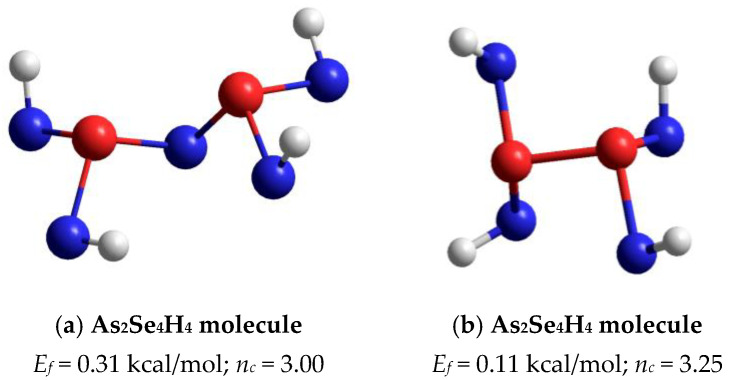
The ball-and-stick presentation of H-atom saturated molecular prototypes of network-forming clusters derived from As_4_Se_5_ thioarsenide-type molecule by breaking in four equivalent Se atom positions: (**a**) As_2_Se_4_H_4_ molecule reconstructed from As_2_Se_3_ network-forming cluster; (**b**) As_2_Se_4_H_4_ molecule reconstructed from As_2_Se_4/2_ cluster. The terminated H atoms are grey-colored, Se and As atoms are blue- and red-colored, respectively, and bonds between atoms are denoted by colored sticks, respectively.

The optimized ball-and-stick presentation of this As_4_Se_5_ molecule, reconstructed with the help of CINCA modeling [16], is depicted in Figure 5b. This cage-like thioarsenide molecule is composed of four small rings (two pentagons and two hexagons) built of ten heteronuclear (As-Se) bonds and one homonuclear (As-As) bond in the under-constrained topology of C_2v_ symmetry (in view of *n_c_*~2.89). In contrast to the above As_4_Se_6_ molecule, the forming energy of this one (As_4_Se_5_) is better, approaching *E_f_*~0.32 kcal/mol. However, molecular crystalline species do not exist in binary As-Se system at this composition, presumably because of competitive iso-compositional network-forming clusters, which can be stabilized as derivatives from As_4_Se_5_ molecules by breaking in all available Se atom positions (like in other thioarsenide-type molecular entities [7,8,38]). Following the concept of preferential molecular network disproportionality [7,8,38], the most plausible among such derivatives (As_4_Se_5_ clusters) is that formed by quadruple x4-breaking in four equivalent Se atom positions. This cluster labeled as x4-As_4_Se_5_ is composed of two separate parts (individual network clusters), these being As_2_Se_3_ representing two corner-sharing AsSe_3/2_ pyramids (which can be derived by quadruple x4-breaking from As_4_Se_6_ thioarsenide-type molecule, x4-As_4_Se_6_ [16]) and As_2_Se_4/2_ representing homonuclear (As-As) covalent bonds in the environment of four Se atoms (which can be derived by quadruple breaking from As_4_Se_4_ molecule, x4-As_4_Se_4_ [38]). The ball-and-stick presentation of H-atom saturated molecular prototypes of these network-forming clusters are, respectively, reproduced in Figure 6a,b.

**Figure 7:**
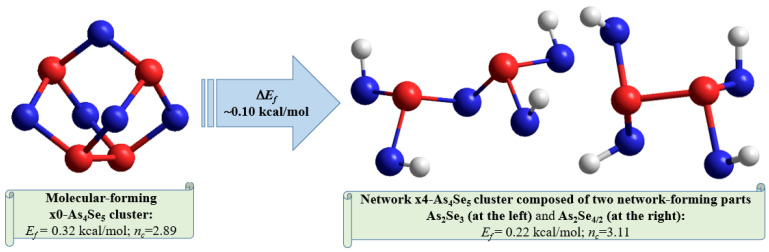
The ball-and-stick presentations of intrinsic decomposition scenario governing molecular-to-network disproportionality in g-As_4_Se_5_. The thioarsenide-type As_4_Se_5_ molecule is transformed by quadruple breaking in four equivalent Se atom positions in a network cluster consisting of two network-forming parts (As_2_Se_3_ and As_2_Se_4/2_). The optimized configurations of H-atom saturated molecular prototypes of these clusters are reproduced with Se and As atoms labeled by blue- and red-colored balls and terminated H atoms labeled by grey balls (see text for more details).

Overall, the optimized configuration of a network-forming x4-As_4_Se_5_ cluster composed of two separate parts (As_2_Se_3_ and As_2_Se_4/2_ network-forming clusters, see Figure 6) is stress-rigid (over-constrained in view of *n_c_* approaching 3.11), while quite energetically favorable and comparable to As_4_Se_5_ thioarsenide-type molecule (x0-As_4_Se_5_), possessing the forming energy *E_f_* approaching 0.22 kcal/mol. This finding means that the glass-forming ability of binary arsenoselenides near a tetraarsenic pentaselenide composition (As_4_Se_5_) is defined by *intrinsic decomposition* of respective molecular-forming clusters (x0-As_4_Se_5_) on network-forming ones equivalent to boundary compositions (x4-As_4_Se_6_ and x4-As_4_Se_4_), but not in other molecular-forming entities (such as x0-As_4_Se_6_ [16] and x0-As_4_Se_4_ [38] clusters), thereby obeying molecular-to-network disproportionality scenario with the barrier Δ*E_f_* =0.10 kcal/mol (see also Figure 7):

(x0-As_4_Se_5_) → (x4-As_4_Se_5_) = [As_2_Se_3_ + As_2_Se_4/2_]. (Δ*E_f_* = +0.10 kcal/mol) (1)

Under increased As content beyond As_4_Se_5_ composition, the number of neighboring As_2_Se_4/2_ groups grows abnormally, accelerating molecular-forming tendencies in a binary As-Se system and resulting in arsenoselenides of higher *molecularity* due to x0-As_4_Se_4_ molecular-forming entities (see Figure 5c) and their low-order network-forming derivatives, such as x1-As_4_Se_4_ clusters [38].

Under nanomilling, products of disproportionality reaction (1) are stabilized, resulting in a great number of molecular x0-As_4_Se_4_ clusters with respect to the following scenario:

(x4-As_4_Se_5_) → As_2_Se_3_ + (x0-As_4_Se_4_), (Δ*E_f_* = +0.04 kcal/mol) (2)

or their most energetically favorable network-forming derivatives (such as x4-As_4_Se_4_ and/or x1-As_4_Se_4_ clusters [38]) with respect to the following scenarios:

(x4-As_4_Se_5_) → As_2_Se_3_ + (x4-As_4_Se_4_), (Δ*E_f_* = +0.17 kcal/mol) (3)

(x4-As_4_Se_5_) → As_2_Se_3_ + (x1-As_4_Se_4_). (Δ*E_f_* = +0.11 kcal/mol) (4)

The network-forming clusters generated under reactions (3) and (4) enhance *reamorphization* tendencies in glassy arsenoselenides compositionally approaching As_4_Se_5_ subjected to nanomilling. By accepting nearly barrier-free stabilization of x0-As_4_Se_4_ molecules in these glasses with respect to reaction (2), it should be suggested that energy of mechanical processing is spent on direct destruction of newly reconstructed x0-As_4_Se_4_ molecules followed by incorporation of their remnants in As-Se glass network.

## 3. Materials and Methods

### 3.1. Preparation of Glassy Arsenoselenides and Their Mechanical Activation

The glassy samples of stoichiometric arsenic triselenide (g-As_2_Se_3_) and thioarsenide-type glass alloys deviated from this stoichiometry towards tetraarsenic pentaselenide (g-As_4_Se_5_) and tetraarsenic tetraselenide (g-As_4_Se_4_) were fabricated by vibrational melt-quenching method from elemental precursors (the As and Se of 5N purity stored in protective argon atmosphere), as described in more detail elsewhere [5,6,7,8]. The sealed ampoules filled with As and Se were placed in a rocking furnace, heated to 925 K, and homogenized for 10 h. Then, they were cooled to 775 K and quenched in water. To eliminate residual stress possible in glassy ingots under rapid cooling, they were preliminarily annealed for 1 h at ~400 K. At the final stage, the ingots extracted from the ampoules were completely amorphous as this follows from their XRD patterns showing diffuse peak-halos typical for amorphous substances. The macroscopic densities of the examined arsenoselenides defined by the Archimedes displacement method in ethanol were in good agreement with the known counterparts from the As-Se system [1,2].

The prepared glassy alloys were subjected to nanomilling in a high-energy planetary ball mill Pulverisette 6 (Fritsch GmbH, Weimar, Germany), transforming preliminary prepared coarse-grained pieces of the glasses sieved under 200 μm (~3g) in the fine-grained powder. Mechanical activation was achieved in this mill for 60 min under a protective Ar atmosphere with 500 min^−1^ rotational speed in a 250 mL tungsten carbide chamber loaded with 50 tungsten carbide balls (each ~10 mm in diameter).

Under such mechanical milling conditions, the energy transfer to the fine-grained powder estimated through specific grinding work performed in the mill of this kind was ~300–320 kJ/g [5,6,7,8].

### 3.2. Medium-Range Structural Changes in Molecular Network Arsenoselenides

The medium-range structure of glassy arsenoselenides was recognized with powder XRD analysis employing the STOE STADI P (STOE & Cie GmbH, Darmstad, Germany) diffractometer operational in transmission mode (Cu Kα_1_-radiation), as described in more detail elsewhere [5,6,7,8].

The amorphous phase was identified by parameterizing diffuse peak-halos in the XRD patterns of the glasses; in part, the FSDP was a signature of IRO-forming structural entities, and the SSDP was a signature of ERO-forming structural entities.

The arrangement of diffuse peak-halos in the XRD patterns responsible for the amorphous phase was analyzed using the STOE WinXPOW 3.03 [39] and PowderCell 2.4 [40] program packages, following the normalization procedure with respect to the maximum of these diffuse peak-halos. The error bar in the diffuse peak-halo position (*θ*) and full width at half maximum (FWHM) were not worse at ±0.05°2*θ*; the scattering vector and width were defined as *Q* = (4π/*λ*)·sin*θ* and Δ*Q* = (4π/*λ*)·sin(FWHM/2). The characteristic distance *R* (the spacing of peak-halo responsible quasi-periodicity) and correlation length *L*, over which this quasi-periodicity was maintained, were calculated as the 2π-reciprocal of the above *Q* and Δ*Q* parameters (*R* = 2π/*Q* and *L* = 2π/Δ*Q*).

Within the modified microcrystalline model [5,6,7,8], the diffuse peak-halos arrangement in the XRD patterning of glassy arsenoselenides was also interpreted as arising from the diffraction of coordination spheres, i.e., the closest inter-atomic (inter-molecular) distances like in randomly-packed multiparticulate systems [41], when experimental XRD patterning is governed by the known Ehrenfest relation [42]:

2*d_s_*·sin *θ* = 1.23·λ, (5)

where *d_s_* is used for the *average inter-atomic distance* between scatterers (the radius of the coordination sphere).

Notably, the error bar in the above linear parameters (*R*, *L*, and *d_s_*) does not exceed ±0.1 Å.

**Figure 8:**
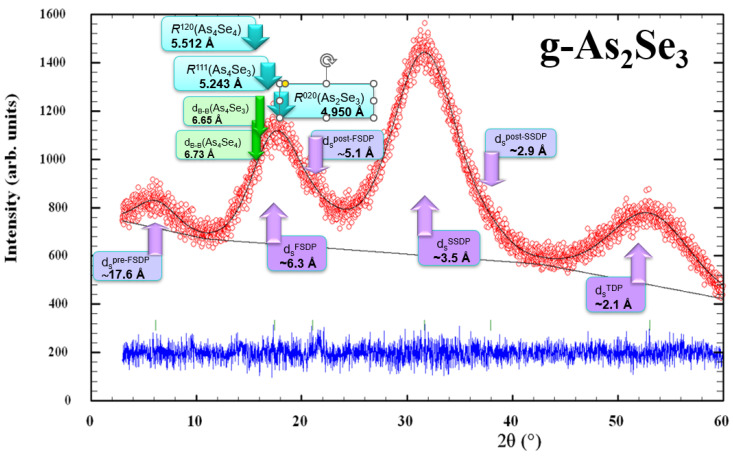
Positioning of experimental (red points) and calculated (black solid line) XRD profiles in melt-quenched samples of g-As_2_Se_3_ showing the arrangement of diffuse peak-halos in respect to most prominent inter-planar and inter-molecular correlations in crystalline As_2_Se_3_, As_4_Se_4_ and As_4_Se_3_ (the difference is reproduced by blue curve at the bottom). The most significant features of this patterning ascribed to inter-molecular correlations are shown by purple-colored arrows, and theoretical features ascribed to crystalline counterparts are shown by the blue- and green-colored arrows.

As an example in favor of a modified microcrystalline model describing the medium-range structure of glassy arsenoselenides [5,6,7,8], Figure 8 demonstrates the positioning of three principal diffuse peak-halos (the FSDP, SSDP, and TDP) along with their most prominent supplements not reproducible in the structure factor determination (such as pre-FSDP, post-FSDP and post-SSDP) in unmilled samples of stoichiometric g-As_2_Se_3_ in respect to most prominent inter-planar correlations in the known arsenoselenide crystalline counterparts, such as *R*(120) = 5.512 Å in monoclinic As_4_Se_4_ [19,20,21], *R*(111) = 5.243Å in orthorhombic As_4_Se_3_ [22], and *R*(020) = 4.950 Å in monoclinic As_2_Se_3_ [18,19]. It also determines *correlations* between barycenters of thioarsenide-type molecules (*d*_B-B_) forming dense molecular packing in crystalline As_4_Se_4_ and As_4_Se_3_ (approaching respectively 6.73 Å and 6.65 Å [7,8]).

It is clearly seen (even from visual inspection of Figure 8) that the FSDP positioning in stoichiometric arsenic triselenide (g-As_2_Se_3_) is defined preferentially by network correlations related to inter-layer spacing in monoclinic As_2_Se_3_ *R*(020) = 4.950 Å. In contrast, molecular correlations due to the dense packing of thioarsenide-type molecules in As-rich crystalline counterparts (monoclinic As_4_Se_4_ [19,20,21] and orthorhombic As_4_Se_3_ [22]) are more decisive in the low-angular shifting and narrowing of the FSDP under transition to over-stoichiometric As-rich arsenoselenides.

### 3.3. Atomic-Specific Microstructure Response on Polyamorphism in Glassy Arsenoselenides

The microstructure of glassy arsenoselenides was tested by micro-RS spectroscopy using the Horiba Xplora spectrometer (Horiba Ltd., Kyoto, Japan) equipped with a CCD detector operational at room temperature (see [8] for more details). The CW 785 nm laser of 90 mW power was used for excitation, with a 10% power option being used to avoid photostructural effects. The spectral resolution was maintained at 2 cm^−1^, and the spatial resolution was near 2 μm. The number of scans was chosen depending on the surface of the sample to be sure that micro-RS spectra processed with Horiba LabSpec software 6 were identical. Unmilled and milled samples were compared through normalization by matching the spectral areas of interest. The RS-active bands were identified using available data for analogs [35,36].

Thermoanalytical responses on *polyamorphism* in glassy arsenoselenides were studied using the multifrequency temperature-modulated DSC-TOPEM^®^ method using the DSC-1 calorimeter (Mettler-Toledo, Greifensee, Switzerland). In this method, the stochastic temperature modulations were superimposed on the underlying rate of DSC scans, resulting in frequency-dependent and independent phenomena, providing more information on the stability of the revealed phases [29,30]. The DSC-TOPEM^®^ instrument was equipped with an FRS5+ sensor and HT100 (Huber, Offenburg, Germany) intracooler; the STAR^e^ ver.13a software was used to control conditions and process the data. The calorimeter was multi-point calibrated using standard probes. The tested samples were encapsulated in sealed 40 μL Al pans kept in a N_2_ atmosphere, scanned at a 1.0 K·min^−1^ rate, and stochastically modulated in 0.75 K pulses between 20 s and 60 s. The evaluations were adjusted using a sapphire reference curve, the width and shift of the calculation window being, respectively, 60 s and 1 s. The heat transfer events were parameterized using the DSC-TOPEM^®^ profiles, presenting temperature variations of non-reversing (*HF*_nrev_) and reversing (*HF*_rev_) heat flow in a heating run; each protocol was averaged in triplicate. In accord with [31,32], the reversing effects due to second-order transitions were parameterized by heat capacity variations Δ*C*_p,_ allowing glass-transition temperatures in onset (*T*_g_^onset^) and mid-point (*T*_g_^mid^) determination. In contrast, non-reversing effects due to enthalpy relaxation in the glass-transition region under heating were parameterized by specific enthalpies difference Δ*H.*

### 3.4. Atomic-Deficient Probing of Molecular Network Disproportionality in Glassy Arsenoselenides

The method of PAL spectroscopy was used as a probe of atomic-deficient free-volume entities in molecular network glassy arsenoselenides [15]. The PAL spectra were recorded with the fast-fast coincidence system ORTEC (230 ps in resolution) using a ^22^Na isotope in normal measuring statistics (~1 M coincidences). The best fitting of the PAL spectra was achieved with the LT 9.0 program [43] under decomposition into three exponentials obeying normalization (*I_1_* + *I_2_* + *I_3_* = 1); model-independent average positron lifetime *τ_av_*^Σ^ was defined as a mass center of the three-component spectrum. Employing this fitting procedure, the raw PAL spectra can be reconstructed with an error bar in lifetimes *τ_i_* and component intensities *I_i_* at the level approaching ±0.005 ns and 0.5%, respectively.

Such an approach allows adequate description of multi-channel PAL spectra in nano-structured substances arising from positrons annihilating in (i) defect-free bulk states, (ii) intrinsic trapping sites (such as vacancies or vacancy-type voids, pores, clusters), and (iii) Ps-hosting free-volume holes employing different mathematical algorithms based on canonical STM [44,45]. In nanostructured substances with a slight contribution from the third component (as in dry-nanomilled arsenoselenides), one of the most plausible seems to be a two-state simple-trapping model ignoring Ps-decaying [15]. Within this approach, the positron-trapping modes (defect-specific *τ_2_* and defect-free bulk *τ_b_* lifetimes, positron trapping rate κ*_d_*, and a fraction of trapped positrons *η*) can be parameterized as in [44,45]. The ‘remainder’ over positron trapping is ascribed to Ps-decaying input owing to positrons annihilating as free particles or interacting with electrons from the environment [46]. Thereby, the Ps localized in holes give an indication of their radii *R* in terms of *τ_3_* lifetime in respect to the semiempirical Tao–Eldrup equation with some material constants, and fractional free volume of Ps-hosting holes (*f_v_*) is defined accepting *I_3_* intensity and empirical constant validated for materials without groups inhibiting Ps formation.

### 3.5. Cluster Modeling of Molecular and Network Structural Conformations in Arsenoselenides

The optimized configurations of thioarsenide As_4_Se_n_-type molecules and network derivatives reconstructed from these molecules by breaking on separate fragments interlinked by Se_1/2_…Se_1/2_ bridges were reconstructed using ab initio quantum-chemical atomic cluster-modeling code CINCA [16]. The HyperChem Release 7.5 program based on the restricted Hartree–Fock self-consistent field method with split-valence double-zeta basis set and single polarization function 6-311G* [47,48,49] was used. Geometrical optimization and single-point energy calculations were performed using the Fletcher–Reeves conjugate gradient method until the root mean square gradient of 0.1 kcal/(Å·mol) was reached. The cluster-forming energy (*E_f_*) was corrected on the energy of terminated H atoms transforming network-forming cluster in a molecule [50] and recalculating this energy with respect to the energy of single AsSe_3/2_ pyramid (*E_f_* = −72.309 kcal/mol [16]).

This method [16] allows the simulation of both molecular and network clusters in covalent systems like glassy arsenoselenides characterized by different coordination numbers (CN). To compare clusters accounting for small rings character for molecular thioarsenides As_4_Se_n_, the number of Lagrangian constraints per atom *n_c_* was calculated using the Phillips–Thorpe constraint-counting algorithm with stretching and bending forces ascribed to intra-molecular bonds within the cluster [51,52,53].

## 4. Conclusions

Polyamorphic transitions driven by high-energy mechanical milling (nanomilling) are recognized in thioarsenide As_4_Se_n_-type glassy alloys deviated from arsenic triselenide As_2_Se_3_ stoichiometry towards over-stoichiometric arsenoselenides compositionally approaching tetraarsenic pentaselenide g-As_4_Se_5_ and tetraarsenic tetraselenide g-As_4_Se_4_, employing the multi-experimental approach based on powder X-ray diffraction (XRD) analysis complemented by thermophysical heat-transfer, micro-Raman scattering (micro-RS) spectroscopy and positron annihilation lifetime (PAL) studies. The microstructure scenarios of nanomilling-driven transformations in arsenoselenides are identified by ab initio quantum-chemical modeling using the authorized cluster modeling code CINCA (Cation-Interlinked Network Cluster Approach).

A straightforward interpretation of the medium-range structural response on milling-driven polyamorphic transitions in the examined arsenoselenides is developed within a modified microcrystalline model treating diffuse peak-halos in their XRD patterning as a superposition of the Bragg-diffraction contribution from some inter-planar correlations, supplemented by the Ehrenfest-diffraction contribution from most prominent inter-atomic/molecular correlations related to derivatives of As_2_Se_3_-type and molecular As_4_Se_4_ -type network-forming structures. The medium-range structural changes in glassy arsenoselenides subjected to nanomilling occur as an interplay between the disrupted intermediate-range and enhanced extended-range ordering. Domination of network conformations in glasses near As_2_Se_3_ stoichiometry (such as g-As_4_Se_5_) results in slight changes in heat transfer and micro-RS responses. At the atomic-deficient level, these transformations are accompanied by a reduced trapping rate of positrons in agglomerated free-volume defects such as multiatomic vacancies in an amorphous As-Se network. Under further increases in As content beyond over-stoichiometric g-As_4_Se_5_ approaching g-As_4_Se_4_, nanomilling-driven responses are essentially enhanced due to the higher molecularity of this glass enriched in thioarsenide As_4_Se_4_ molecules and their low-order network derivatives.

## Figures and Tables

**Table 1:** Parameterization of the FSDP-related diffuse peak-halo in unmilled and nanomilled glassy arsenoselenides under deviation from As_2_Se_3_ stoichiometry in As-rich side.

Glassy Arsenoselenides		The FSDP Position		The FSDP Width		Characteristic Distance	Correlation Length	Interatomic Distance
Composi-tion, ref.	state	*θ*, *°2θ*	*Q*_1_, Å^−1^	Δθ, *°2θ*	Δ*Q*_1_, Å^−1^	*R,* Å	*L,* Å	*d_s_*, Å
g-As_2_Se_3_, [6]	unmilled	18.014(13)	1.28	6.02(4)	0.43	4.92	14.67	6.05
	milled	17.862(11)	1.27	6.02(3)	0.43	4.96	14.65	6.10
g-As_4_Se_5_, this work	unmilled	16.771(10)	1.19	4.20(3)	0.30	5.28	21.02	6.50
	milled	17.104(16)	1.21	5.41(5)	0.38	5.18	16.33	6.37
g-As_4_Se_4_, [7]	unmilled	16.130(8)	1.14	3.21(2)	0.23	5.49	27.52	6.75
	milled	16.608(13)	1.19	4.55(4)	0.32	5.33	19.42	6.56

**Table 2:** Parameters of the DSC-TOPEM profiles in unmilled and nanomilled arsenoselenides g-As_x_Se_100-x_ derived from reversing (*HF*_rev_) and non-reversing (*HF*_nrev_) heat flow variations.

Glassy Arsenoselenide As_x_Se_100-x_ Sample		Derived from *HF*_rev_			Derived from *HF*_nrev_
		Glass-Transition Temperature		Heat Capacity Variation	Specific Enthalpies Difference
composition	state	*T*_g_^onset^, ^o^C	*T*_g_^mid^, ^o^C	Δ*C*_p_, J·g^−1^K^−1^	Δ*H*, J·g^−1^
g-As_2_Se_3_	unmilled	179.0	187.4	0.20	6.7
	milled	178.3	185.9	0.20	−10.9
g-As_4_Se_5_	unmilled	183.9	190.0	0.17	3.8
	milled	182.0	188.7	0.17	−9.6

**Table 3:** The best-fit PAL spectra parameterization for unmilled and nanomilled arsenoselenides under small deviation from As_2_Se_3_ stoichiometry.

Glassy Arsenoselenides: Composition, State (Sample)	[FIT-1]	*τ_1_*, ns	*τ_2_*, ns	*τ_3_*, ns	*I_2_*, a.u.	*I_3_*, a.u.	*τ_av_.*, ns
g-As_2_Se_3_, unmilled (bulky discs)	0.01	0.193	0.358	2.091	0.62	0.007	0.310
g-As_2_Se_3_, nanomilled (pellets)	0.05	0.202	0.371	2.087	0.54	0.015	0.322
g-As_4_Se_5_, unmilled (bulky discs)	0.02	0.196	0.359	1.858	0.62	0.008	0.310
g-As_4_Se_5_, nanomilled (pellets)	0.06	0.204	0.373	2.204	0.52	0.015	0.325
g-As_4_Se_4_, unmilled (bulky discs)	0.05	0.205	0.370	1.982	0.55	0.015	0.322
g-As_4_Se_4_, nanomilled (pellets)	0.05	0.210	0.378	2.264	0.52	0.013	0.325

**Table 4:** The best-fit PAL spectra trapping modes for unmilled and nanomilled glassy arsenoselenides under small deviation from As_2_Se_3_ stoichiometry determined within a two-state simple trapping model ignoring Ps-decay contribution.

Glassy Arsenoselenides: Composition, State (Sample)	Positron-Trapping MODES					Ps-Decay Modes	
	*τ_b_*, ns	*κ*_d_, ns^−1^	*τ_2_-τ*_b_, ns	*τ_2_/τ*_b_, a.u.	*η*, a.u.	*R_3_*, nm	*f_v_^3^*, %
g-As_2_Se_3_, unmilled (bulky discs)	0.272	1.51	0.09	1.32	0.29	0.296	0.15
g-As_2_Se_3_, nanomilled (pellets)	0.270	1.24	0.10	1.37	0.25	0.296	0.29
g-As_4_Se_5_, unmilled (bulky discs)	0.273	1.45	0.09	1.32	0.28	0.275	0.12
g-As_4_Se_5_, nanomilled (pellets)	0.272	1.23	0.10	1.37	0.25	0.306	0.32
g-As_4_Se_4_, unmilled (bulky discs)	0.272	1.23	0.10	1.23	0.25	0.306	0.32
g-As_4_Se_4_, nanomilled (pellets)	0.274	1.12	0.10	1.38	0.23	0.311	0.30

## Data Availability

The original contributions presented in this study are included in the article. Further inquiries can be directed to the corresponding author.
